# Utilization of Hospital Room Hospitality Features on Patient-Controlled Tablet Computers: Cohort Study

**DOI:** 10.2196/13964

**Published:** 2019-06-20

**Authors:** Beiqun Zhao, Ming Tai-Seale, Christopher Longhurst, Brian Clay

**Affiliations:** 1 Department of Surgery University of California San Diego La Jolla United States; 2 Department of Family Medicine and Public Health University of California San Diego La Jolla, CA United States; 3 Department of Pediatrics University of California San Diego La Jolla, CA United States; 4 Department of Medicine University of California San Diego La Jolla, CA United States

**Keywords:** inpatients, electronic health records, patient satisfaction, patients’ rooms

## Abstract

**Background:**

Patient portals tethered to electronic health records can improve patient experience, activation, and outcomes. However, adoption of inpatient portals has been challenging. One way to potentially increase inpatient portal usage is to integrate it with a room control (RC) app on a common tablet computer.

**Objective:**

The aim of this study was to perform a retrospective analysis of patient usage of an RC app provided on tablet computers in patient rooms of our new inpatient tower.

**Methods:**

We identified all patients who were admitted for >24 hours to our new inpatient tower over a 90-day period from September 1 to November 30, 2017. After excluding newborn patients from our analysis, we then identified patients who used the RC app at least one time during their admission. We linked these data to patient demographics (including age, sex, and race) and admitting service. We then performed univariable and multivariable logistic regression to assess patterns of RC app usage.

**Results:**

A total of 3411 patients were admitted over the course of the study period; 2242/3411 (65.73%) used the RC app during their hospitalization. Compared with white patients, other/mixed/unknown race and Asian, Hawaiian, Pacific Islander, American Indian race were significantly associated with increased use of the RC app in a multivariable analysis. Increasing age was significantly associated with increased usage of the RC app. Usage of the RC app also varied by admitting services. Compared with general medicine, bone marrow transplant and general surgery patients had increased usage of the RC app. Conversely, critical care, medical specialties, neurology, surgical subspecialties, and obstetrics/gynecology were all associated with decreased usage of the RC app.

**Conclusions:**

Our study shows that one-third of patients are not using the RC app for critical room functions. Future initiatives to increase RC usage should take these populations into consideration. Contrary to common belief, older patients may use tablet-enabled RCs just as often, if not more often, than younger patients. Certain admitting services, such as neurology and surgical subspecialties, may have had lower usage rates owing to accessibility issues. Our study allows hospitals to tailor support for specific patient populations to increase RC app usage.

## Introduction

Electronic health records with patient portals allow patients to conveniently access their health information, which can improve patient experience, patient activation, and patient care [1-3]. Although outpatient portals are becoming more common, challenges persist in the widespread adoption of inpatient portals [4]. However, it has been shown that colocation of the inpatient portal with a room control (RC) app on a tablet can increase patient utilization of the inpatient portal [5] and may increase patient engagement [6].

In addition, RC apps can centralize frequently used and critical room functions (eg, lighting, curtains, and television controls), thereby enabling patients to better control their environment while being confined to a hospital bed. This has led some to consider RC apps as a hospitality feature for patients, which may be a point of contention in today’s health care environment owing to increased concerns over hospital spending on nonclinical amenities [7]. However, hospitality features have been associated with improved patient satisfaction [8,9], which can potentially affect hospital reimbursement [10,11]. In addition, hospitality features and improved patient satisfaction can lead to better patient outcomes [12-15]. For example, empowering patients with RC apps may reduce calls to nursing staff for room comfort needs, which can decrease patient call-light burden, leading to improved patient care [16].

However, it is not known which patients are more or less likely to use RCs. Answering this question will allow hospitals to tailor support for specific patient populations to increase RC usage. In this study, we have explored our initial experience with implementing an RC app on tablets in patient rooms at our institution.

## Methods

**Figure 1:**
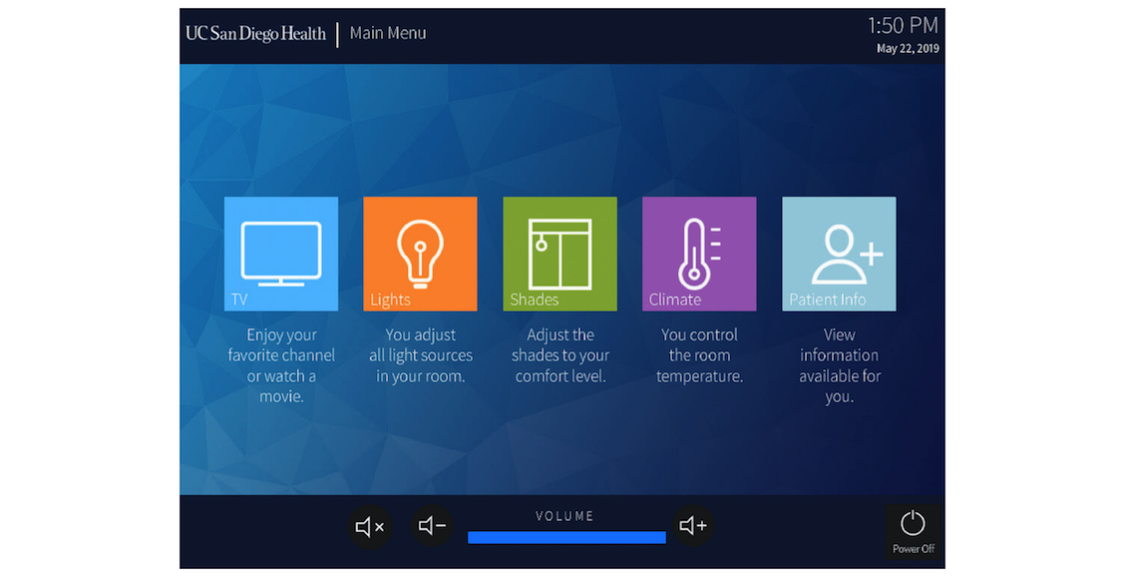
The main menu of the room control app.

**Figure 2:**
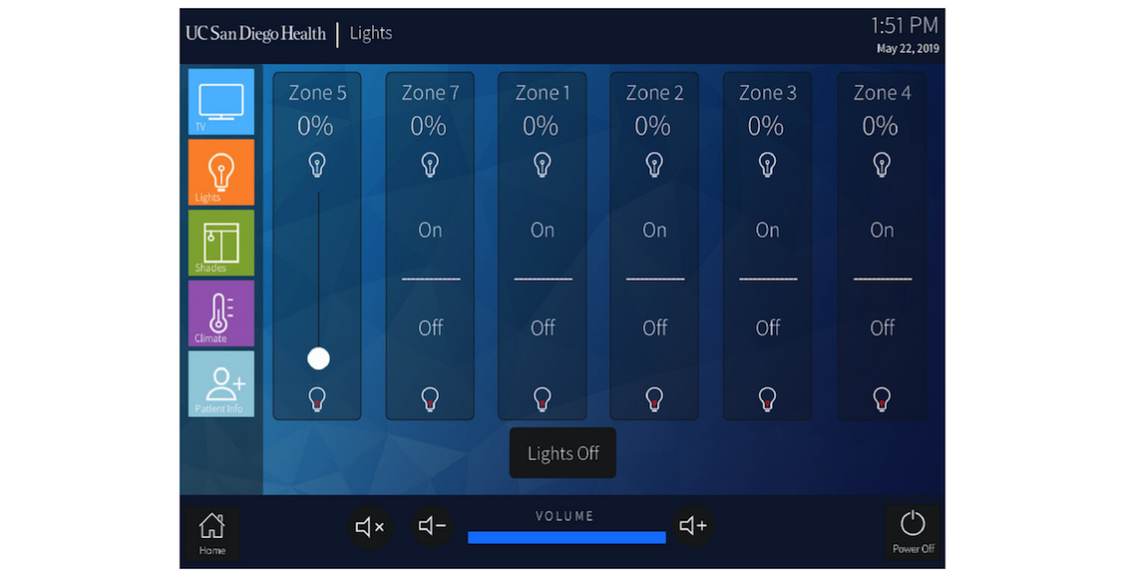
The lighting control submenu of the room control app.

In designing the patient rooms for our new inpatient tower, our institution made the conscious decision to integrate multiple RCs (eg, curtains, lighting, and television controls) into a central patient-facing app (Crestron Electronics) installed on tablet computers (Apple Inc) in every patient room (Figures 1 and 2).

We sought to examine patient factors associated with using RC features on the tablet. After obtaining institutional review board exemption, we analyzed data over a 90-day period from September 1 to November 30, 2017, for all hospital admissions of >24 hours. We examined the proportion of patients who accessed the RC app during their hospitalization by linking RC usage data with patient admission data. After patient linkage, we were able to collect patient demographic information, including sex, age, and race. We also identified the length of stay and admitting service for each patient. This was then categorized into general medicine (including family medicine, internal medicine, and hospitalist services), bone marrow transplant, critical care (including surgical and medical critical care services), general surgery (including transplant, surgical oncology, colorectal, vascular, minimally invasive, and plastic surgery services), medical specialty (including cardiology, pulmonary, medical oncology, and gastroenterology services), neurology (including stroke and neurology services), obstetrics and gynecology, and surgical subspecialty (including urology, head and neck, neurosurgery, and orthopedic surgery services). We excluded newborn patients from our analysis. We performed the Mann-Whitney U test and univariable and multivariable logistic regression analysis using IBM SPSS Statistics (IBM Corp, version 25.0). The level of significance was set at .05 for all analyses.

## Results

During our study period, 3411 patients were identified. Of these patients, 2242/3411 (65.73%) patients used the RC app at some point during their hospitalization. A comparison of sex, age, race, and admitting service is shown in Table 1. Patients who used the RC app had significantly longer hospital length of stay than patients who did not use the RC app (*P*<.001). Univariable and multivariable logistic regression is shown in Table 2. In a multivariable analysis, other/mixed/unknown race and Asian, Hawaiian, Pacific Islander, American Indian (AHPIAI) race were associated with increased use of the RC app (odds ratio [OR]=1.27; *P*=.008 and OR=1.51; *P*=.006, respectively) compared with white patients. In addition, increasing age was associated with increased usage of the RC app. Compared with general medicine, bone marrow transplant and general surgery patients had increased usage of the RC app. Conversely, critical care, medical specialty, neurology, surgical subspecialty, and obstetrics/gynecology were all associated with decreased usage.

**Table 1:** Patient demographics stratified by room control app usage (N=3411).

Patient demographic		Used RC^a^ app	Did not use RC app
Total number of patients, N (%)		2242 (65.73)	1169 (34.27)
**Sex, n (%)**			
	Female	1542 (68.78)	949 (81.18)
	Male	700 (31.22)	220 (18.82)
**Race, n (%)**			
	White	1286 (57.36)	723 (61.85)
	Black	97 (4.33)	49 (4.19)
	Asian, Hawaiian, Pacific Islander, American Indian	216 (9.63)	82 (7.01)
	Other/Mixed/Unknown	643 (28.68)	315 (26.95)
**Age (years), n (%)**			
	16-30	365 (16.28)	337 (28.83)
	31-45	740 (33.00)	449 (38.41)
	45-65	651 (29.04)	208 (17.79)
	>65	486 (21.68)	175 (14.97)
**Admission Service, n (%)**			
	General medicine	378 (16.86)	97 (8.30)
	Medicine specialty	14 (0.62)	65 (5.56)
	General surgery	419 (18.69)	37 (3.17)
	Surgical subspecialty	246 (10.97)	124 (10.61)
	Bone marrow transplant	228 (10.17)	13 (1.11)
	Critical care	27 (1.20)	51 (4.36)
	Neurology	70 (3.12)	43 (3.68)
	Obstetrics/Gynecology	860 (38.36)	739 (63.22)
Hospital length of stay, median (range)		5 (1-492)	2 (1-205)

^a^RC: room control.

**Table 2:** Univariable and multivariable logistic regression.

Variables		Univariable analysis		Multivariable analysis	
		*P* value	Odds ratio (95% CI)	*P* value	Odds ratio (95% CI)
Female		<.001	0.511 (0.430-0.606)	.28	—^a^
**Race**					
	White	—	Reference	—	Reference
	Black	.555	—	.31	—
	Asian, Hawaiian, Pacific Islander, American Indian	.004	1.481 (1.130-1.940)	.006	1.510 (1.129-2.020)
	Other/Mixed/Unknown	.097	—	.008	1.274 (1.064-1.526)
**Age (years)**					
	16-30	—	Reference	—	Reference
	31-45	<.001	1.522 (1.260-1.838)	<.001	1.494 (1.225-1.822)
	45-65	<.001	2.890 (2.330-3.583)	.002	1.591 (1.179-2.149)
	>65	<.001	2.564 (2.042-3.219)	.006	1.586 (1.140-2.204)
**Admission service**					
	General medicine	—	Reference	—	Reference
	Medicine specialty	<.001	0.055 (0.030-0.103)	<.001	0.036 (0.018-0.071)
	General surgery	<.001	2.906 (1.9414.350)	<.001	3.288 (2.186-4.944)
	Surgical subspecialty	<.001	0.509 (0.3730.694)	.001	0.588 (0.427-0.808)
	Bone marrow transplant	<.001	4.501 (2.4668.215)	<.001	3.288 (2.186-4.944)
	Critical care	<.001	0.136 (0.081-0.228)	<.001	0.104 (0.060-0.180)
	Neurology	<.001	0.418 (0.269-0.649)	.001	0.472 (0.302-0.739)
	Obstetrics/Gynecology	<.001	0.299 (0.234-0.381)	<.001	0.490 (0.352-0.681)
Hospital length of stay		<0.001	1.056 (1.043-1.069)	<.001	1.040 (1.025-1.054)

^a^Not applicable.

## Discussion

### Principal Findings

We found that 65.7% of patients admitted to our new inpatient tower used an integrated RC app installed on tablet computers inside patient rooms. After controlling for other predictors, we found that the AHPIAI race and other/mixed/unknown race was associated with increased RC app use. Surprisingly, we also found that increasing age was associated with *increased* RC app use. In addition, longer hospital length of stays were predictive of RC app usage. Finally, compared with general medicine, patients admitted to general surgery and bone marrow transplant had more RC app use, whereas patients admitted to medicine specialty, surgical subspecialty, critical care, neurology, and obstetrics/gynecology all had less RC app use than general medicine.

There has been growing concerns that elderly patients may be disadvantaged by the influx of technology in health care today [17]. However, in our study, we found that as age increased, use of the RC app also increased. This may be partly explained by the intuitive nature of the tablet interface, and previous literature has shown success in using tablet-based apps in elderly patients [18]. Furthermore, this finding showcases that RC apps can potentially function as a gateway for elderly patients to access other patient-facing technology.

### Secondary Findings

Another interesting finding is the large variation in RC use among admitting services. Patients admitted to the bone marrow transplant service may have had the highest rate of RC app usage because these patients are often admitted to the hospital for prolonged periods of time. However, even after controlling for length of stay, these patients were still more likely to use the RC app. This is most likely because their movement in and out of their unit is limited owing to their disease process and are thereby more likely to explore hospitality features. Increased use of the RC app in general surgery patients may be because these patients are often confined to their hospital beds after surgery and are unable to control the blinds or lights through the usual physical switches on the wall. In this case, the RC app gives patients more control over their environment, which may improve the patient experience during a highly vulnerable time. This pattern is not seen in surgical subspecialty patients, potentially because these patients, especially neurosurgery, head and neck surgery, and orthopedic surgery patients, may not be physically able to operate a tablet computer after surgery. This may also explain the decreased usage of RCs in neurology patients. Therefore, further work will be needed to increase accessibility in these patients (perhaps through voice-enabled features). Patients admitted to the critical care service are clinically very sick, which may limit their usage of ancillary technology such as tablet computers. Similarly, medicine subspecialty patients may also represent a group of patients with high clinical acuity. Obstetrics and gynecology represent a very diverse group of patients, making interpretations difficult with regard to their usage of the RC app. Future studies will focus on barriers to RC app usage unique to each admitting service and exploring potential ways to increase RC app usage for each service.

### Limitations

Given its retrospective nature, we are unable to ensure that RCs were used by the patient and not by a family member. However, RC app use by family members may still encourage use of other apps found on the tablet. It is also possible that the RC app was used by the nursing staff to demonstrate the RCs to the patient, though we suspect this occurrence is rare. Interpretation of use patterns of obstetrics and gynecology patients was difficult because they represent a diverse group of patients, with different indications and acuity. This service includes patients admitted to labor and delivery for observation and patients undergoing postoperative care after a large cancer operation. The former group may be more similar to general medicine patients, whereas the latter group may be more similar to general surgery patients. Unfortunately, we are unable to separate the different obstetrics/gynecology patients in the current analysis. Although RC apps may increase inpatient portal usage [5], we are unable to track the usage of other apps on the tablet at this time. In addition, we do not have granular data of RC app usage, such as average daily use length and specific features accessed. Obtaining this type of data may require a prospective analysis (ie, installing a tracking software) and will be a source of future studies. In addition, patient-level data, such as income and education, were not available. These factors may play a role in the adoption of the RC apps.

### Conclusions

Our study shows that approximately one-third of patients are not using the RC app to control critical room functions, and future initiatives to increase RC app usage should take these populations into consideration. Despite its intuitive interface, there may still be accessibility limitations to the current RC app, especially for patients admitted to certain services. A more thorough exploration of why the RC app usage is low in these patient populations is needed in the future.

## Abbreviations

AHPIAI: Asian, Hawaiian, Pacific Islander, American Indian
OR: odds ratio
RC: room control
